# Combination of p53AIP1 and survivin expression is a powerful prognostic marker in non-small cell lung cancer

**DOI:** 10.1186/1756-9966-28-22

**Published:** 2009-02-19

**Authors:** Shin-ichi Yamashita, Masao Chujo, Michiyo Miyawaki, Keita Tokuishi, Kentaro Anami, Satoshi Yamamoto, Katsunobu Kawahara

**Affiliations:** 1 Department of Surgery II, Faculty of Medicine, Oita University, 1-1 Idaigaoka, Hasama, Yufu, Oita, 879-5593, Japan

## Abstract

**Background:**

p53AIP1 is a potential mediator of apoptosis depending on p53, which is mutated in many kinds of carcinoma. High survivin expression in non-small cell lung cancer is related with poor prognosis. To investigate the role of these genes in non-small cell lung cancer, we compared the relationship between p53AIP1 or survivin gene expression and the clinicopathological status of lung cancer.

**Materials and methods:**

Forty-seven samples from non-small cell lung cancer patients were obtained between 1997 and 2003. For quantitative evaluation of RNA expression by PCR, we used Taqman PCR methods.

**Results:**

Although no correlation between p53AIP1 or survivin gene expression and clinicopathological factors was found, the relationship between survivin gene expression and nodal status was significant (p = 0.03). Overall survival in the p53AIP1-negative group was significantly worse than in the positive group (p = 0.04); however, although survivin expression was not a prognostic factor, the combination of p53AIP1 and survivin was a significant prognostic predictor (p = 0.04). In the multivariate cox proportional hazard model, the combination was an independent predictor of overall survival (p53AIP1 (+) survivin (+), HR 0.21, 95%CI = [0.01–1.66]; p53AIP1 (+) survivin (-), HR 0.01, 95%CI = [0.002–0.28]; p53AIP1 (-) survivin (-), HR 0.01, 95%CI = [0.002–3.1], against p53AIP1 (-) survivin (+), p = 0.03).

**Conclusion:**

These data suggest that the combination of p53AIP1 and survivin gene expression may be a powerful tool to stratify subgroups with better or worse prognosis from the variable non-small cell lung cancer population.

## Introduction

A number of genes for apoptosis play an important role in tumorigenesis [1]. Several gene abnormalities were reported as prognostic markers of non-small cell lung cancer, such as p53 [2]; however, these processes are complex and remain unclear.

The abnormal expression of p53 is frequently reported in a variety of cancers [3]. p53 mutations are generally more common in smokers than in nonsmokers and an excess of G to T transversions of p53 has been described as a molecular signature of tobacco smoke mutagens in smoking-associated lung cancers. There are also mutational hotspots (codons 157, 158, 245, 248, and 273) in the p53 gene in lung cancer [4]. Several reports have shown that p53 expression is a prognostic marker in non-small cell lung cancer [2]. p53 protein is a tumor suppressor gene and mediates cell cycle arrest or programmed cell death [5,6]. These p53-mediated events were triggered through the transactivation of specific genes, including p21, GADD45, cyclin G1, Bax, and fas [7,8].

Recently, we reported that p53AIP1, which is a new potential mediator of p53-dependent apoptosis, is associated with prognosis in non-small cell lung cancer [9]. p53AIP1 is not normally expressed in any tissues except the thymus, but is induced when Ser-46 of p53 was phosphorylated after severe DNA damage [10,11]. Only a few papers have reported p53AIP1 function in cancer biology and it has not been well investigated [9,12].

On the other hand, survivin is a member of the *IAP *gene family, which has been implicated in both the inhibition of apoptosis and mitosis regulation [13]. Survivin up-regulates genes in tumor tissues [14]. High survivin expression in the primary tumor is related to poor prognosis in many cancer types [15-20]. As p53 leads to the repression of survivin expression [21], p53 AIP1 might act inversely against survivin in the same manner as p53. It is interesting to evaluate both the expression of the p53AIP1 gene and survivin in primary non-small cell lung cancer.

In this study, we demonstrated the expression of these genes in non-small cell lung cancer and normal lung tissue, and the combination of p53AIP1 with survivin may be a prognostic marker.

## Methods

### Patients and Samples

This study was approved by the Institutional Review Board of the National Hospital Organization Kumamoto Medical Center (Kumamoto, Japan) and all patients completed informed consent forms. Forty-seven operative samples from non-small cell lung cancer (NSCLC) patients were obtained at the National Hospital Organization Kumamoto Medical Center (Kumamoto, Japan) between May 1997 and September 2003. The samples were histologically diagnosed as primary non-small cell lung cancer according to the WHO classification. None of the cases had received radiation therapy or chemotherapy before surgery. Adjacent normal lung tissue was also taken from all cases. Tissue specimens were frozen immediately with RNA later™(QIAGEN) and stored at -80°C until RNA extraction. RNA from tissue samples was prepared using TRIzol reagents (Invitrogen). To evaluate cigarette consumption, a smoking index (SI) was used: cigarette consumption per day multiplied by smoking years. Referring to this index, smokers were divided into 2 groups, heavy smokers with indices ≥ 400, and light smokers < 400.

### Quantitative PCR analysis

For quantitative evaluation of the RNA expression by PCR, we used Taqman PCR methods (TaqMan^® ^Gene Expression Assays; Applied Biosystems, Tokyo, Japan) as previously reported [22]. The p53AIP1 gene was amplified by the following primer set as follows, reverse: ggggacttctcaggtcgtgt, forward: tggacttcttcatgccccga.

The p53AIP1 gene internal probe was ttgcggtgcgagtcgtggaagtaa. Survivin was amplified by the following primer set: reverse: ggggacttctcaggtcgtgt, forward: tggacttctt catgccccga. The survivin internal probe was ttgcggtgcgagtcgtgg aagtaa.

PCR amplification condition were one cycle of 50°C, 2 min, and 95°C, 10 min followed by 50 cycles of 95°C, 15 sec and 60°C, 1 min. The measured value was calculated by comparative Ct methods [22] and GAPDH gene amplification was used as a control. All reactions were duplicated. The amounts of p53AIP1 and survivin mRNA were expressed as n-fold GAPDH mRNA and the levels were compared relative to adjacent normal lung tissues. A tumor/normal ratio of p53AIP1 and survivin mRNA expression greater than 1 was identified as a positive expression, and the others as negative.

### Statistical analysis

All statistical analysis was performed using Stat View J5.0 (SAS Institute Inc.). Different variables of tumors and normal tissues were analyzed with the chi-square test or Fisher's exact test. Overall survival was analyzed using the Kaplan-Meier method and evaluated by the log-rank test. Significant differences were considered at p < 0.05. The cutoff point was also p < 0.05 for univariate and multivariate Cox proportional hazard model analysis.

## Results

p53AIP1 and survivin expression in primary non-small cell lung cancer (NSCLC) was evaluated by real-time RT-PCR. All 47 samples were studied with paired histopathologically normal lung tissues which were far from the tumor margin. Table 1 shows a correlation between the clinicopathological status and p53AIP1 and survivin gene expressions. Although no relationship between the p53AIP1 gene expression and variables (age, sex, smoking index (SI), tumor size, nodal status, histological type) was not found, the survivin gene expression-positive rates in the node metastasis-positive group were significantly higher than in the negative group (p = 0.03).

**Table 1:** Correlation between p53AIP1 or survivin expression and clinicopathological characteristics

Characteristics	All patients		p53AIP1 positive	p	Survivin positive	p
Age	<70	19	11		14	
	≥70	28	14	0.23	14	0.45
						
Sex	male	14	6		11	
	female	33	19	0.36	17	0.08
						
Smoking	<400	19	10		13	
index	≥400	28	15	0.95	15	0.31
						
Tumor	T1	27	16		18	
	T2	16	9		8	
	T3	4	0	0.08	2	0.52
						
Nodal status	N0	33	12		10	
	N1	14	5	0.17	9	0.03*
						
Histologic type	Ad	27	12		19	
	Sq	16	10		7	
	others	4	3	0.34	2	0.22

Ad, adenocarcinoma; Sq, squarmous cell carcinoma

* statistically significant

**Figure 1:**
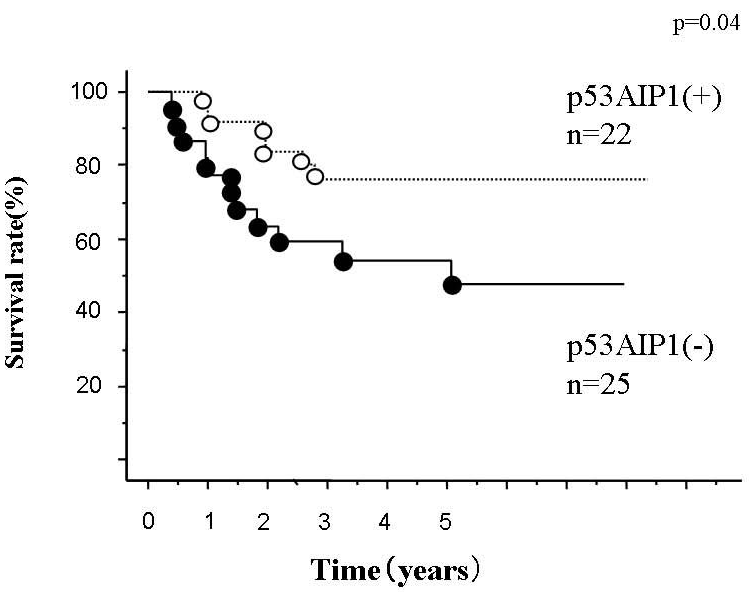
**Overall survival curves according to p53AIP1 gene expression**. Differences are significant (p = 0.04). Number of patients in each group, positive, 22; negative, 25.

**Figure 2:**
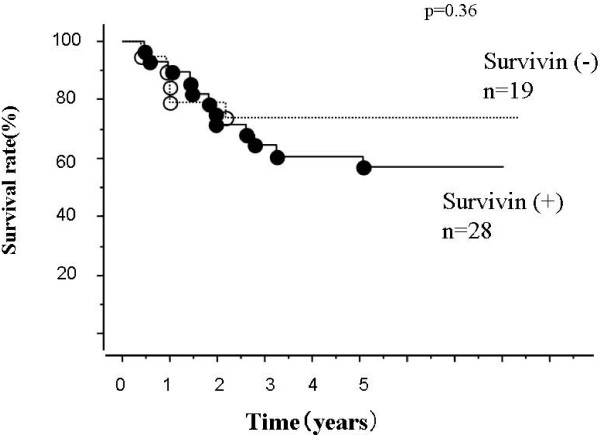
**Overall survival curves according to survivin gene expression**. Differences are not significant (p = 0.36. Number of patients in each group, positive, 28; negative, 19.

**Figure 3:**
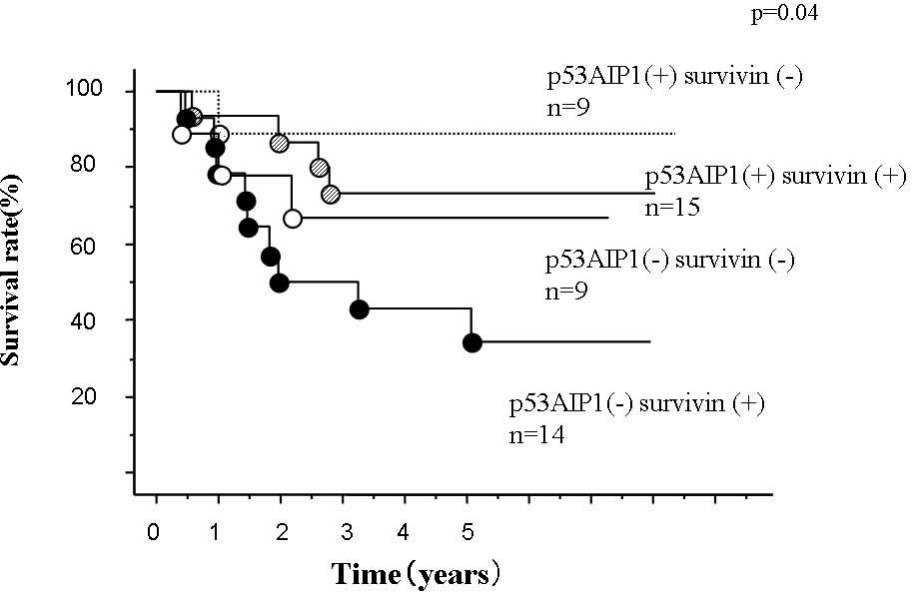
**Overall survival curves according to the combination of p53AIP1 and survivin gene expression**. Differences are statistically significant (p = 0.04). Number of patients in each group, p53AIP1 positive and survivin positive, 15; p53AIP1 positive and survivin negative, 9; p53AIP1 negative and survivin positive, 14; p53AIP1 negative and survivin negative, 9.

Figure 1 shows the overall survival curves by Kaplan-Meier analysis for patients with non-small cell lung cancer classified according to p53AIP1 expression (positive, tumor/normal ratio ≥ negative, <1). Patients in the positive p53AIP1 expression group have a better prognosis than the negative expression group (p = 0.04). The median follow-up period was 5.4 years (1.2 to 8.4 years); however, the superiority of the survivin expression negative group to the positive group for overall survival was not significant (Figure 2). When we compared the prognosis according to the variable combination between p53AIP1 and survivin, the p53AIP (+) survivin (-) group had the best prognosis (Figure 3). In contrast, the p53AIP (-) survivin (+) group showed the worst prognosis and the other two groups were intermediate. In univariate analysis using age, tumor size, lymph node metastasis, histological type, survivin expression, p53AIP1 expression, and the combination of p53AIP1 and survivin, p53AIP1 and the combination were statistically significant (Table 2).

**Table 2:** Clinicopathological factors and p53AIP1 or survivin expression for overall survival in univariate and multivariate Cox regression analysis

Characteristics		Univariate analysis		Multivariate analysis	
		HR (95%CI)	p	HR (95%CI)	p
Age	<70	1	0.55		0.86
	≥70	1.34 (0.52–3.48)			
					
Tumor	T1	1	0.63		0.93
	T2	1.08 (0.14–8.58)			
	T3	1.72 (0.21–14.0)			
					
Nodal status	N0	1	0.47		0.89
	N1	1.46 (0.52–4.17)			
					
Histologic type	Ad	1	0.23		0.06
	Sq	0.41 (0.11–1.49)			
	others	0.28 (0.06–1.25)			
					
survivin	(+)	1	0.36		0.19
	(-)	0.62 (0.22–1.75)			
					
p53AIP1	(+)	1	0.04*		0.48
	(-)	2.67 (0.99–7.25)			
					
Combination			0.04*		0.03*
p53AIP1 (-) survivin (+)		1		1	
p53AIP1 (+) survivin (+)		0.31 (0.09–1.0)		0.21(0.01–1.66)	
p53AIP1 (+) survivin (-)		0.12 (0.02–0.97)		0.01 (0.002–0.28)	
p53AIP1 (-) survivin (-)		0.46(0.12–1.7)		0.01(0.002–3.1)	

Ad, adenocarcinoma; Sq, squarmous cell carcinoma

* statistically significant

In multivariate Cox proportional hazard model analysis, the combination (p = 0.03) was an independent predictor of overall survival (Table 2).

## Discussion

The molecular mechanism of tumor progression and apoptosis is still unclear. Several predictors, such as nodal involvement, tumor stage, and survivin and p53 have been reported; however, the relationship between p53 or survivin and the prognosis of lung cancer patients is still controversial [2,23]. As we recently reported, p53AIP1 in primary non-small cell lung caner has a potential role as a prognostic factor [9]. Additionally, the other report showed that truncating variants of P53AIP1 were associated with prostate cancer [12]. A recent report showed that p53AIP1 was directly regulated by not only p53 but p73 [24]. This might be supported by the result which did not show a correlation between p53 mutation and p53AIP1 expression [9], and it may be interesting to investigate the p73 expression with p53AIP1. The present study showed that p53AIP1 is not related to any clinicopathological factors, which is different from the report that p53AIP1 is closely related to nodal status in our previous study [9]. This might be due to different analysis methods, the frequency or quantification of expression levels. Although univariate analysis showed that p53AIP1, a proapoptotic gene, is a good predictor of overall survival despite no correlation with several factors, multivariate analysis did not show this because of the limited sample size.

On the other hand, as previously reported, survivin-positive expression correlated with more aggressive behavior and poorer prognosis [13]. Meta-analysis of the role of survivin showed that positive survivin might be a prognostic factor [23]. In our study, survivin is significantly related to the nodal status, which may suggest that survivin plays the key role in lymph node metastasis, as in the previous report [25]; however, survivin was not a significant prognostic factor in the present study. It is difficult to estimate the prognostic value of survivin because the power of this study was low due to the limited sample size; therefore, we considered the combination of biomarkers to be more powerful to show the prognostic value of survivin and p53 AIP1.

The rationale was as follows, since p53 leads to the repression of survivin expression, and apoptotic cells induced by p53 caused resistance to apoptosis when survivin was overexpressed [21], p53 AIP1 might have an inverse effect against survivin in the same manner as p53. Furthermore, as the relationship between survivin and p53AIP1 has not been investigated, we hypothesized that the combination analysis of survivin with p53AIP1 can be a powerful tool for risk stratification. The combination of negative p53AIP1 and positive survivin showed the worst prognosis, leading to the speculation that these two genes act in an opposite manner and are critical for tumor progression. Multivariate analysis showed that the combination of these genes was an independent predictor of survival. Furthermore, p53AIP1 and survivin expressions in non-small cell lung cancer cells before chemotherapy may contribute as independent predictors of the effect of chemotherapy, such as DNA-damaging agents.

In conclusion, although the sample size was small, our study demonstrated that the combination of survivin with p53AIP1 gene expression in non-small cell lung cancer is a possible independent prognostic factor. Further investigation of these combinations might show the prognostic significance of these genes in non-small cell lung cancer.

## Abbreviations

RT-PCR: reverse transcription-PCR.

## Competing interests

The authors declare that they have no competing interests.

## Authors' contributions

Authors have made substantial contributions to conception and design (MC, MM, and SY), acquisition of data (KT and KA), analysis, interpretation of data, organizing study (SY), and supervision of research group (KK)
